# Influence of Triploid *Musa* spp. Genome Background and Exogenous Growth Regulators on In Vitro Regeneration in Plantains and Bananas

**DOI:** 10.3390/plants14142109

**Published:** 2025-07-09

**Authors:** Labode Hospice Stevenson Naitchede, Onyinye C. Ihearahu, Kishan Saha, David O. Igwe, Supriyo Ray, George Ude

**Affiliations:** Department of Natural Sciences, Bowie State University, 14000 Jericho Park Road, Bowie, MD 20715, USA; lnaitchede@bowiestate.edu (L.H.S.N.); oihearahu@bowiestate.edu (O.C.I.); ksaha@bowiestate.edu (K.S.); digwe@bowiestate.edu (D.O.I.); sray@bowiestate.edu (S.R.)

**Keywords:** *Musa* spp., genomes, biotic stress, abiotic stress, planting material, plant growth regulators, shoot induction, rooting

## Abstract

Bananas and plantains, belonging to the *Musa* genus, are important food crops that sustain the livelihoods of countless smallholder farmers globally. However, their production is hindered by various challenges, including abiotic and biotic stresses, climate change, and poor access to clean planting materials, which negatively impact their yields. Addressing these constraints is essential for improving production and ensuring food security. This study investigated the influence of triploid genome background and exogenous growth regulators on the regeneration of *Musa* cultivars [Gros Michel (AAA genome), Obino l’Ewai and Silk (AAB genome), and Poteau Naine (ABB genome)]. Shoot tip explants of the AAA, AAB, and ABB triploid genomes were cultured in Murashige and Skoog (MS) media supplemented with varying 6-benzylaminopurine (BAP) and indole-3-butyric acid (IBA), indole-3-acetic acid (IAA), or naphthaleneacetic acid (NAA) hormones. Shoot induction was successfully achieved within 21.50 ± 2.00 days, with AAA exhibiting the highest shoot induction frequencies ranging from 30.00 ± 1.57% to 100% and shoot numbers per explant ranging from 3.00 ± 0.50 to 8.80 ± 0.80, followed by the ABB genome ranging from 20.00 ± 3.45% to 100% and from 2.00 ± 0.55 to 5.60 ± 0.50 shoots, and the AAB genome ranging from 17.50 ± 5.01% to 100% and from 2.00 ± 0.04 to 6.60 ± 0.25 shoots, respectively, in media amended with 1.2 to 6.0 mg.L^−1^ BAP and 0.1 mg.L^−1^ IAA. The highest rooting rate of 100% was recorded in all three genomes in media containing 1.4 mg.L^−1^ IBA and 0.5 mg.L^−1^ IAA, with the AAA genome producing the maximum number of 14.8 roots per explant. The results indicate the positive influence of the AAA genome background on in vitro regeneration and its potential utilization for genomic editing transformation protocols

## 1. Introduction

Bananas (*Musa sinensis* L.) and plantains (*Musa paradisiaca* L.) belong to the *Musaceae* family and are the world’s second-largest fruit crops, with an annual production of 130 million metric tons [1,2]. They are essential food sources and cash crops in tropical and subtropical regions, mainly in developing countries, holding great significance in global trade [3]. For instance, in Sub-Saharan Africa, bananas and plantains are important food crops that sustain the livelihoods of countless smallholder farmers. West Africa alone is a key area for plantain cultivation globally, contributing approximately 32% to the total production around the world [3,4]. In terms of imports and exports, bananas and plantains are the most traded fruit globally, and the USA is the lead banana-importing country, making up over 19% of the world’s total [5]. Banana import in the USA peaked in 1999 and has since fluctuated between 3800 and 4100 thousand tons annually, leading to large enterprises cultivating bananas in Central America to meet the growing demand at the start of the 20th century [6]. In addition, the distribution of metabolites in bananas and plantains is crucial for their nutritional and therapeutic benefits, as they are rich in fiber, potassium, vitamins, and antioxidants. The decline in nutritional health among high school students over the past two decades is linked to inadequate fruit consumption, prompting the WHO to emphasize food security and nutrition improvement. Enhancing global fruit supply is essential for addressing under-nutrition and obesity [7,8,9,10,11].

However, banana and plantain cultivation in major regions faces biotic and abiotic stresses, limited genetic diversity in germplasm, and insufficient access to clean planting material for smallholder farmers and their livelihoods [12]. Drought is a significant abiotic stress leading to 65% yield losses [13]. Under drought conditions, the growth of banana and plantain plants is inhibited, and fruit yield is significantly reduced, highlighting climate change’s need for drought mitigation efforts. Pests and diseases, particularly Fusarium wilt, Sigatoka leaf spots, bacterial wilt, and virus-induced banana bunchy top and streak virus, pose significant biotic constraints to both banana and plantain production despite their limited geographic distribution [14]. Moreover, the B genome of plantain naturally hosts sequences of *Banana streak virus* (BSV) named endogenous BSV (eBSV). Under drought stress, eBSV produces an episomal virus that spreads as an infection [15]. Factors affecting banana production and livelihoods are exacerbated by triploid cultivars, which pose challenges to breeding programs and genetic diversity expansion efforts.

Though these constraints and environmental factors undermine the development of the banana and plantain value chain, there is potential for increasing yields. Developing resistant cultivars through plant tissue culture is the most effective approach to controlling biotic and abiotic stresses [15,16,17]. Plant propagation techniques like tissue culture are commonly used for the commercial multiplication of several species, with micropropagation being the preferred method for producing thousands of clones from a single plant part through direct organogenesis [18].

Although the effect of plant growth regulators in shoot regeneration has been reported in many monocots and dicots [19,20], very few studies on plant growth regulators have been conducted on banana and plantain plants, particularly those in which cytokinins and auxins are combined to induce plant growth and rooting. Critical hormonal substances, such as gibberellins, cytokinins, and auxins, are commonly used alone in some banana (*Musa*) species for the shoot regeneration with a low shoot formation frequency [21,22]. Furthermore, to date, there is no report on the influence of the triploid *Musa* spp. genome background on the regeneration of banana and plantain.

Therefore, developing an efficient reproducible method for regenerating *Musa* spp., besides mass multiplication and conservation, is fundamental and crucial for initiating transformation protocols for genomic editing towards producing drought-resistant varieties, commercial field production, and dissemination of improved hybrids. Direct organogenesis has been leveraged in the last decade to enhance the in vitro regeneration methods of numerous species. This study explored how the triploid genome background and exogenous growth regulators influence the regeneration of *Musa* cultivars through direct organogenesis, aiming to mass produce elite cultivars with triploid genomes and create drought-resistant varieties to mitigate the water-deficient climate challenges.

## 2. Results

### 2.1. Effect of Exogenous Plant Growth Regulators Alone on Days to Shoot Induction

**Figure 1:**
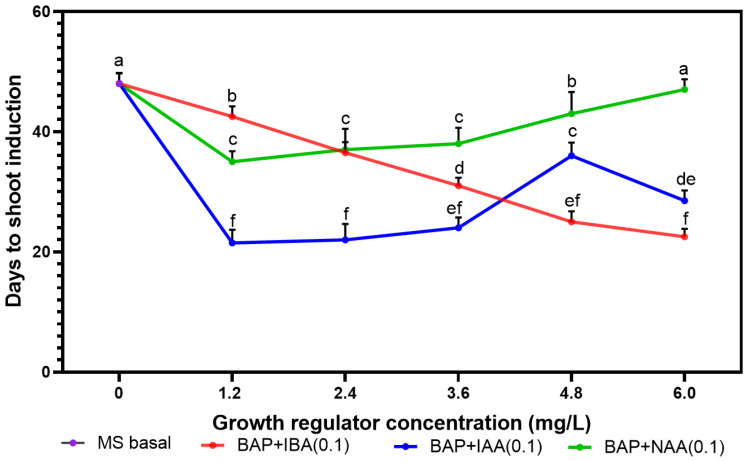
Effect of BAP, IAA, NAA, and IBA on days to shoot formation from *Musa* spp. cultivar shoot tip explants (Gros Michel, Obino l’Ewai, Silk, and Poteau Naine). Values are the mean ± S.E. of 4 replicates per treatment, and all the experiments were conducted thrice. There is no difference between values with the same letter when compared with Fisher’s Least Significant Difference method. (*p* < 0.05); LSD (0.05) = 3.87.

Exogenous growth regulators were used to rebalance endogenous ones. Shoot buds were formed from shoot tip explants on MS media supplemented with 6-benzylaminopurine (BAP), indole-3-acetic acid (IAA), and naphthaleneacetic acid (NAA). The plant growth regulator (PGR) types and concentrations impacted the number of days required for the explant to induce shoots. The days to first shoot formation increased with the increase in PGR concentration from 1.2 to 4.8 mg.L^−1^, except when BAP was combined with IBA, where the number of days to shoot bud formation was inversely proportional to the PGR concentration. However, the time to shoot induction significantly decreased with a further increase in IAA concentration. The shortest time to shoot induction (21.5 ± 2.00 days) was observed on MS medium containing 1.2 mg.L^−1^ BAP and 0.1 mg.L^−1^ IAA (Figure 1), but there was no significant difference with 2.4 mg.L^−1^ IAA and 6.0 mg.L^−1^ IBA. The most significant duration for shoot induction was recorded for MS medium devoid of PGRs (48 days) and 6.0 mg.L^−1^ BAP augmented with 0.1 mg.L^−1^ NAA (47 days).

### 2.2. Effect of Varying Plant Growth Regulators on Adventitious Shoot Formation, Multiplication, and Elongation Irrespective of the Genomes

**Figure 2:**
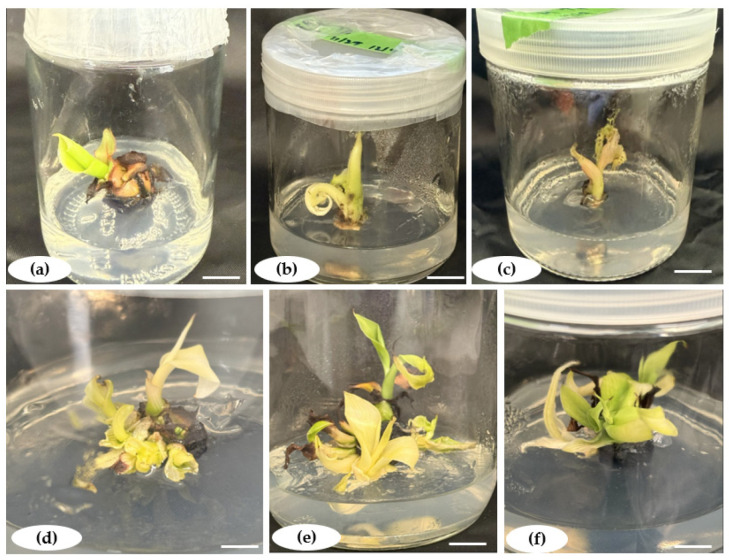
Shoot formation from shoot tip explants of *Musa* spp. on MS medium supplemented with 3% (*w*/*v*) sucrose and 3.6 mg.L^−1^ BAP + 0.1 mg.L^−1^ IAA. (**a**–**c**) represent induced shoots from Gros Michel (AAA), Poteau Naine (ABB), and Silk (AAB), respectively. (**d**–**f**) represent multiple shoot formations from Gros Michel, Poteau Naine, and Silk, respectively. Scale bar = 1.5 cm.

Shoot buds derived from banana and plantain shoot tip explants (Figure 2) were induced on MS media supplemented with different amounts of BAP (0.00–6.0 mg.L^−1^) combined with either IBA, IAA, or NAA (0.1 mg.L^−1^). The efficiency varied depending on the PGRs and quantity applied. The shoot induction rate increased with the BAP concentration (0 to 3.6 or 4.8 mg.L^−1^) irrespective of the type of combination. Above these concentrations of BAP, fewer shoots formed. IAA with BAP was the most suitable combination, producing the highest shoot formation frequency (100%) and, significantly, the largest number of shoots (7 shoots/explant) recorded on MS supplemented with 3.6 mg.L^−1^ BAP combined with 0.1 mg.L^−1^ IAA. Among the different BAP combinations with the other auxins, 4.8 mg.L^−1^ BAP was most efficient when combined with 0.1 mg.L^−1^ NAA or 0.01 mg.L^−1^ IBA, producing a shoot formation frequency of 77.66 ± 2.06% and 50 ± 1.76%, respectively. However, the combination of BAP and 0.1 mg.L^−1^ IBA resulted in lower shoot induction rates than 0.1 mg.L^−1^ IAA and 0.1 mg.L^−1^ NAA mg.L^−1^ (Table 1).

**Figure 3:**
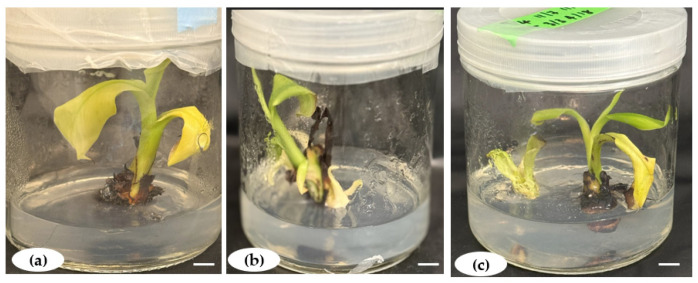
Elongation of in vitro shoots of *Musa* spp. on MS medium with 4.8 mg.L^−1^ BAP and 0.1 mg.L^−1^ IAA. (**a**) Gros Michel (AAA). (**b**) Poteau Naine (ABB). (**c**) Silk (AAB). Scale bar = 1.5 cm.

**Figure 4:**
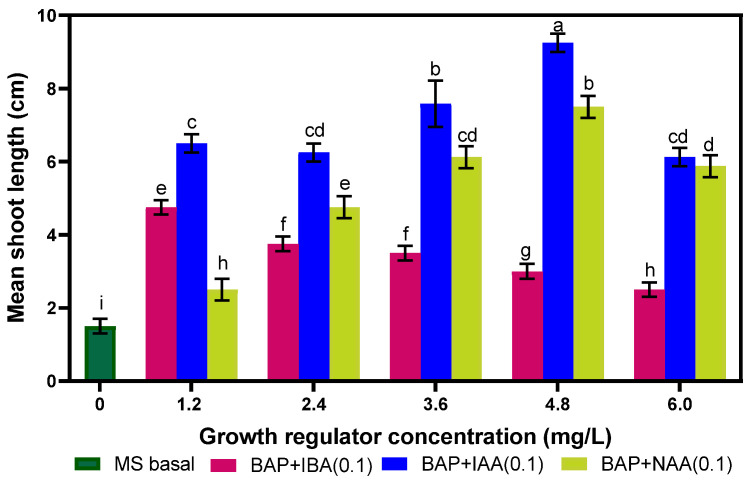
Effect of PGRs alone on shoot elongation from regenerated shoots. No difference between values with the same letter when compared using Fisher’s Least Significant Difference method. (*p* < 0.05); LSD (0.05) = 0.48.

The shoots elongated on the same MS media. The length of elongated shoots rose with increasing PGR concentrations up to 4.8 mg.L^−1^ but declined thereafter, except when BAP was augmented with IBA. The largest shoot height (9.25 ± 0.25 cm) was recorded when 4.8 mg.L^−1^ BAP was combined with 0.1 mg.L^−1^ IAA in the MS media. The smallest elongated plantlet (1.5 ± 0.20 cm) was found in the control media (Figure 3 and Figure 4).

### 2.3. Effect of Triploid Musa spp. Genomes on Adventitious Shoot Proliferation, Multiplication, and Elongation

**Figure 5:**
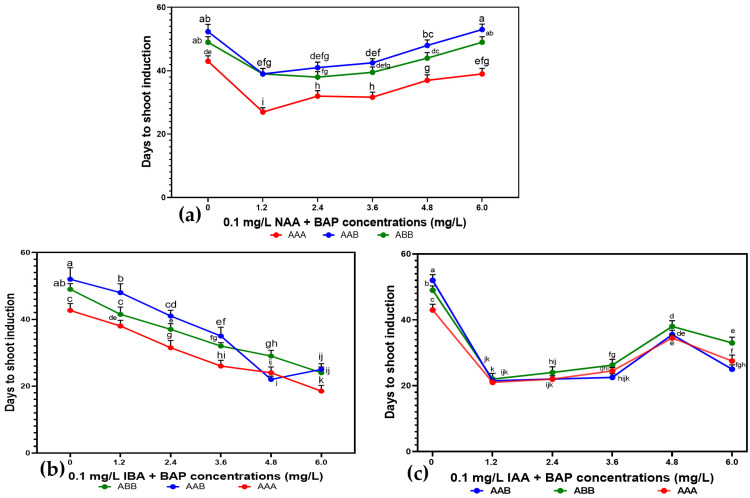
Effect of *Musa* spp. genomes on days to shoot formation from shoot tip explants in MS media supplemented with different BAP concentrations combined with 0.1 mg.L^−1^ NAA (**a**), 0.1 mg.L^−1^ IBA (**b**), and 0.1 mg.L^−1^ IAA (**c**). No difference between values with the same letter when compared with Fisher’s Least Significant Difference method. (*p* < 0.05); LSD (0.05) IBA = 0.49; LSD (0.05) IAA = 0.78; LSD (0.05) NAA = 0.41.

The three *Musa* spp. genomes behaved differently under different PGR formulations with respect to days to first shoot formation. The statistical analysis showed that the genome AAA produced the first shoots in a very short time compared to the other two genomes, with shoots appearing within 27 days, 21 days, and 18.5 days in MS media supplemented with 1.2 mg.L^−1^ BAP + 0.1 mg.L^−1^ NAA, 1.2 mg.L^−1^ BAP + 0.1 mg.L^−1^ IAA, and 6.0 mg.L^−1^ BAP + 0.1 mg.L^−1^ IBA, respectively (Figure 5). The genomes AAB and ABB took longer times (48 and 49 days) to induce shoots with no significant difference with the control medium when the MS media included 1.2 mg.L^−1^ BAP combined with 0.1 mg.L^−1^ IBA, or 6.0 mg.L^−1^ BAP combined with 0.1 mg.L^−1^ NAA, respectively (Figure 5a,b). However, the two genomes (AAB and ABB) exhibited a short time to shoot formation (21.5 ± 1.50 and 22 ± 2.25 days) when MS media was supplemented with 1.2 mg.L^−1^ BAP and 0.1 mg.L^−1^ IAA (Figure 5c) compared to the other two BAP combinations with IBA and NAA.

**Figure 6:**
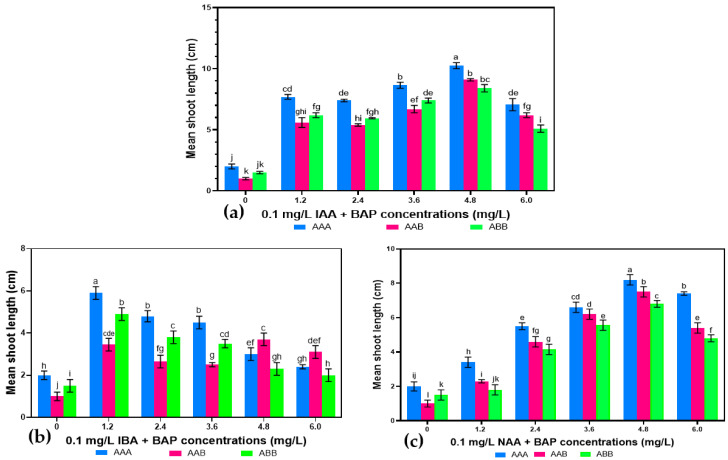
Effect of *Musa* spp. genomes on shoot elongation from regenerated shoots in MS media supplemented with different BAP concentrations combined with 0.1 mg.L^−1^ NAA (**a**), 0.1 mg.L^−1^ IBA (**b**), and 0.1 mg.L^−1^ IAA (**c**). No difference between values with the same letter when compared with Fisher’s Least Significant Difference method. (*p* < 0.05); LSD (0.05) IBA = 0.49; LSD (0.05) IAA = 0.78; LSD (0.05) NAA = 0.41.

The shoot formation frequencies of the three genomes were found to be higher in MS medium augmented with PGR formulations compared to those in the control medium. The increase in the concentration of BAP (up to 4.8 mg.L^−1^) led to an increase in the frequency of shoot induction, whether combined with NAA, IBA, or IAA, whereas the shoot number per explant varied slightly. Among the three genomes tested, the AAA gave significantly higher shoot induction frequencies (from 30% to 100%) and shoot numbers (3 to 8.80) than the genomes AAB (17.5% to 100%; 2 to 6.60 shoots per explant) and ABB (20% to 100%; 2 to 5.60 shoots per explant) in all the tested PGR combinations irrespective of the concentration (Table 2, Table 3 and Table 4). All three genomes produced 100% shoot formation rates in the medium supplemented with 3.6 mg.L^−1^ BAP and 0.1 mg.L^−1^ IAA, with the genome AAA plantlets elongating to 8.6 cm (Figure 6). The genome AAA still performed well when BAP was combined with NAA, producing 100% shoot induction frequency in 3.6 mg.L^−1^ BAP combined with 0.1 mg.L^−1^ NAA. However, the largest shoot lengths found for the genome AAA (10.25 ± 0.25 cm) and AAB (9.1 ± 1.67 cm) were recorded in the medium containing 4.8 0.1 mg.L^−1^ BAP and 0.1 mg.L^−1^ IAA (Figure 6a–c). A small shoot length was noted in MS media devoid of PGRs for all the tested genomes, with the genomes AAB and ABB producing the shortest shoots of 1 ± 0.20 cm and 1.5 ± 0.16 cm, respectively.

### 2.4. Effect of Exogenous Auxins Alone on De Novo Root Formation of Regenerated Plantlets

**Figure 7:**
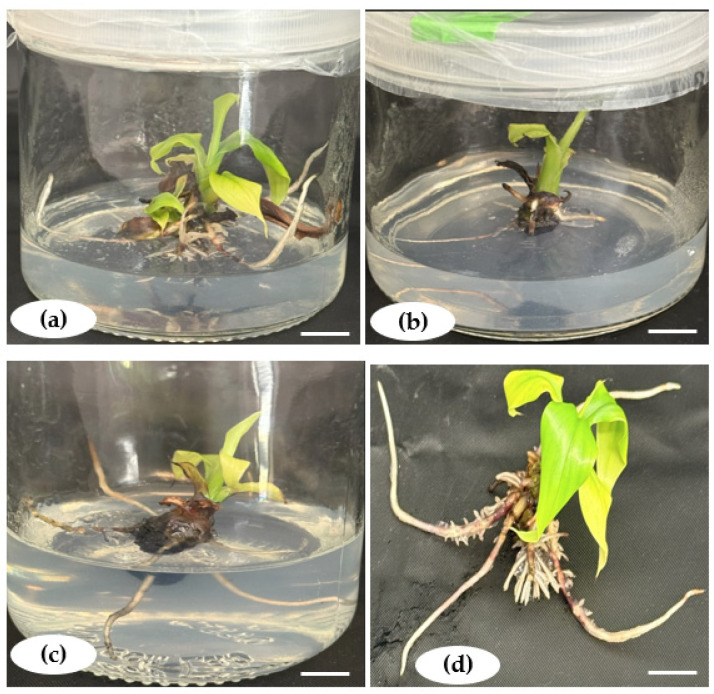
Complete in vitro plantlets of *Musa* spp. (**a**,**d**) Rooting from microshoots of Gros Michel (AAA) and (**b**,**c**) rooting from microshoots of Poteau Naine (ABB) and Silk (AAB), respectively, on MS medium supplemented with 1.4 mg.L^−1^ IBA and 0.5 mg.L^−1^ IAA. Scale bar = 1.5 cm.

**Figure 8:**
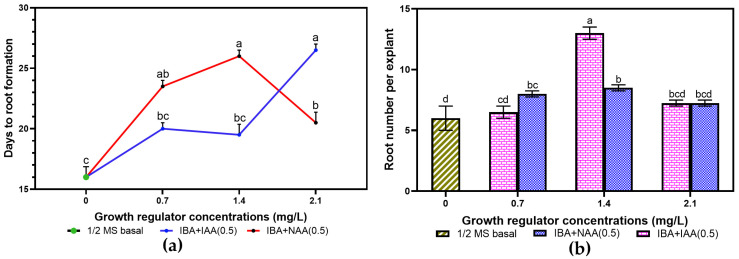
Effect of varying concentrations of IBA combined with 0.5 mg.L^−1^ (IAA or NAA) on root formation of *Musa* spp. (**a**) Days to rooting. (**b**) Root number per microshoot. No difference between values with the same letter when compared with Fisher’s Least Significant Difference method. (*p* < 0.05); LSD (0.05) days to rooting = 4.32; LSD (0.05) root number = 1.70.

Root induction was successfully achieved in all the tested rooting media formulations, with healthy roots sprouting within an average of 20.44 days (Figure 7). However, the days to root formation (DRFs) varied depending on the PGRs and their concentrations (Figure 8a). In media containing IBA combined with 0.5 mg.L^−1^ NAA, the DRFs significantly increased with the IBA concentrations from 16 days to 26 days before decreasing when the IBA concentration was above 1.4 mg.L^−1^. In IBA media formulations with 0.5 mg.L^−1^ IAA, the number of DRFs (16 to 26 days) was proportional to IBA concentrations, with a slight decrease without a significant difference between 0.7 mg.L^−1^ and 1.4 mg.L^−1^ IBA. In total, a short time to root formation was found in hormone-free MS media (negative control), while regenerated shoots took a longer time to produce roots in media with PGRs.

Generally, it was noted that all the regenerated plantlets produced roots. However, there was a very significant difference (*p* < 0.0001) in the parameters evaluated (root formation frequency, number of roots, and root length) among concentrations of BAP and auxins. It was observed that the root induction frequency increased with IBA concentration but significantly reduced beyond 1.4 mg.L^−1^. The maximum root formation frequency, root length and root number were observed when IBA was mixed with IAA in the MS media, with the highest values of 100%, 11.5 ± 0.51 cm, and 13 ± 0.75 roots per plantlet recorded for 1.4 mg.L^−1^ IBA + 0.5 mg.L^−1^ IAA (Table 5 and Figure 8b). MS media without PGRs resulted in a higher root formation rate when compared to the average frequency observed in MS containing IBA combined with NAA. The lowest root length (5.38 ± 0.36 cm) was found in 2.1 mg.L^−1^ IBA and 0.5 mg.L^−1^ NAA, while the same concentration of IBA combined with either 0.5 mg.L^−1^ IAA or NAA produced the smallest root number per explant (7.25 ± 0.25).

### 2.5. Influence of Triploid Musa spp. Genome Background on Root Formation in MS Media, and Plantlets’ Acclimatization

**Figure 9:**
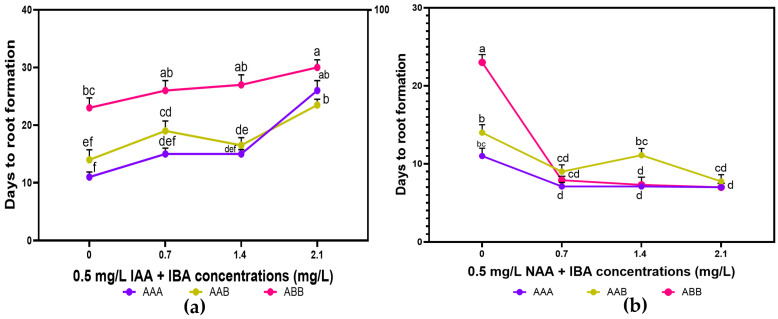
Effect of *Musa* spp. genomes on days to root induction from regenerated plantlets in MS media supplemented with varying IBA concentrations combined with 0.5 mg.L^−1^ IAA (**a**) and 0.5 mg.L^−1^ NAA (**b**). No difference between values with the same letter when compared with Fisher’s Least Significant Difference method. (*p* < 0.05); LSD (0.05) IAA = 4.21; LSD (0.05) NAA = 3.59.

Plantlets of the three genomes responded well and produced roots in media with or without PGRs. The rooting frequency, number of roots, and length were significantly affected by both auxin combinations tested (*p* ≤ 0.004). Though the genome AAA showed better root formation ability in comparison to AAB and ABB (Figure 9), plantlets from the three genomes formed roots faster in MS media containing IBA and NAA (7 to 11 days) than in MS media devoid of PGRs (11 to 23 days) and media supplemented with BAP and IAA (15 to 30 days).

**Figure 10:**
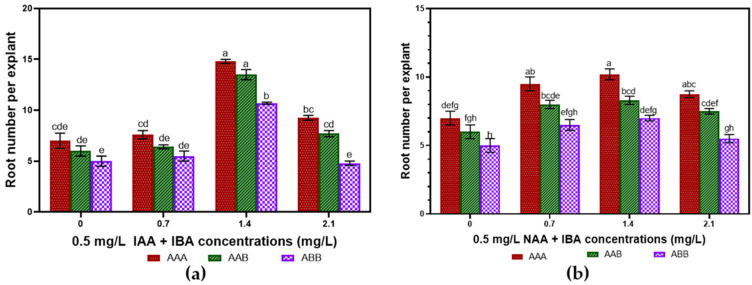
Effect of *Musa* spp. genomes on root number in MS media supplemented with varying IBA formulations with 0.5 mg.L^−1^ IAA (**a**) and 0.5 mg.L^−1^ NAA (**b**). No difference between values with the same letter when compared with Fisher’s Least Significant Difference method. (*p* < 0.05); LSD (0.05) IAA = 2.52; LSD (0.05) NAA = 1.54.

The three genomes showed significantly higher root formation frequencies when MS media contained PGRs than when MS media were devoid of PGRs. Among the three genomes, the AAA exhibited the maximum rooting frequency (80–100%) and more roots per explant (7 to 14.8) irrespective of PGR combination and concentration (Table 6 and Table 7; Figure 10a,b). Plantlets from the genome AAB exhibited better root formation data than the genome ABB, with a rooting rate increasing from 75% to 100% and root number from 6 to 8.3 per explant when they were cultured in MS media containing BAP and IAA, even though, beyond 2.1 mg.L^−1^ IBA, there was less root formation. However, there is no significant difference in root formation frequencies of the three genomes (100%) when they were grown in media supplemented with 1.4 mg.L^−1^ IBA, increased by 0.5 mg.L^−1^ IAA. The three genomes tested in this study showed efficient root induction data in media containing IAA compared to NAA. The genome AAA exhibited the longest root length (12.9 ± 1.53 cm), especially when plantlets were cultured in 1.4 mg.L^−1^ IAA augmented with 0.5 mg.L^−1^ IAA, whereas the AAB produced the shortest root length (3.4 ± 0.51 cm) in media supplemented with 2.1 mg.L^−1^ NAA (Table 7). When regenerated microshoots of the genome ABB were subcultured in the medium amended with 2.1 mg.L^−1^ IBA and 0.5 mg.L^−1^ IAA, they produced the lowest rooting frequency of 45% ± 0.80, which was found to be less than that in the control medium. In addition, the rooting rate obtained in shoots subcultured on the control was higher than the rate in media containing 0.7 mg.L^−1^ IBA and 0.5 mg.L^−1^ NAA, with significant elongation for all the genomes investigated. Moreover, plantlets are more elongated in the control medium than in media supplemented with NAA.

**Figure 11:**
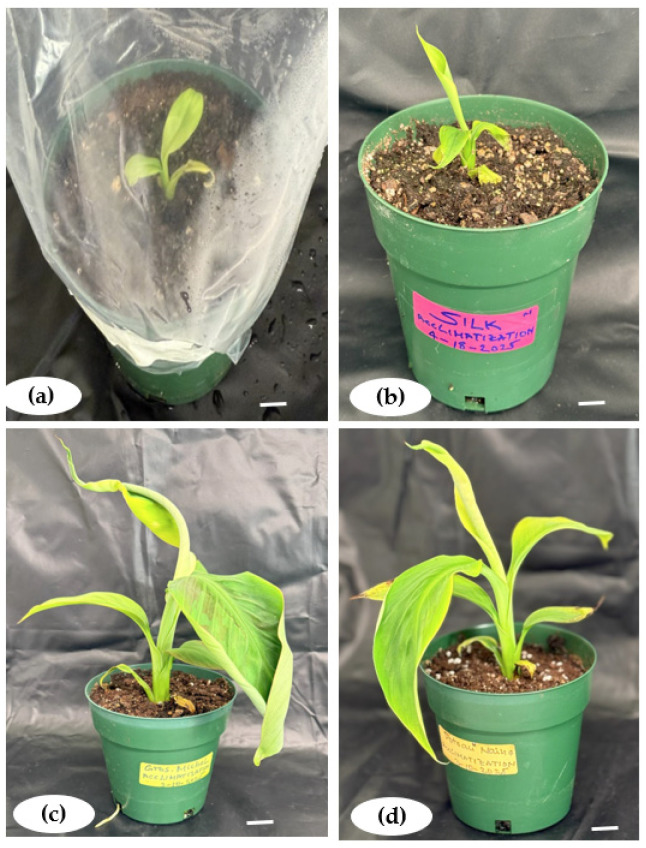
(**a**) Hardened plantlets. (**b**–**d**) Acclimatized plants of Gros Michel (AAA), Poteau Naine (ABB), and Silk (AAB). Scale bar = 2 cm.

The acclimatization approach applied to rooted plantlets was successful. The elongated and rooted plantlets were successfully hardened with the protocol applied (Figure 11a). Acclimatized plants revealed no morphological alterations and appeared normal, with a survival rate of 100% for the three genomes (Figure 11b–d).

### 2.6. Correlation of Plant Growth Regulator Concentration with Shoot Induction and Root Formation

**Figure 12:**
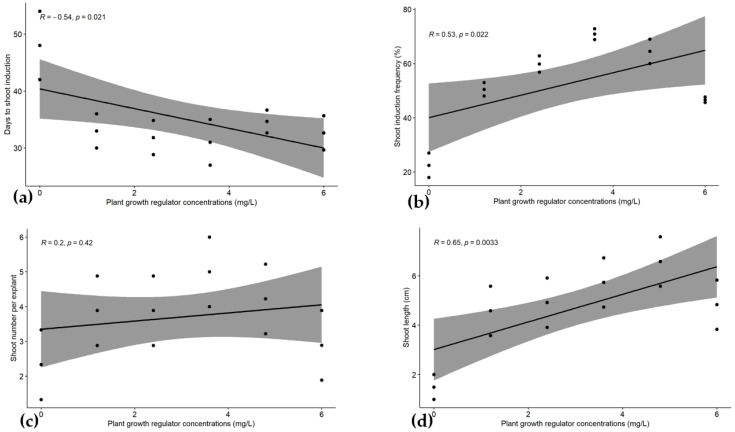
Linear regression curve of plant growth regulator concentrations with days to shoot formation (**a**), shoot induction frequency (**b**), shoot number per explant (**c**), and shoot length (**d**) in *Musa* spp. R = Pearson correlation (R), calculated using RStudio 4.3.2.

The days to shoot formation and shoot induction frequencies showed positive correlation with the BAP concentrations, with Pearson correlation coefficients of r = 0.5 and 0.53, respectively (Figure 12a,b). A stronger correlation was found with shoot length (r = 0.65), but there is no correlation between shoot number and BAP at *p* < 0.05 (Figure 12c,d).

**Figure 13:**
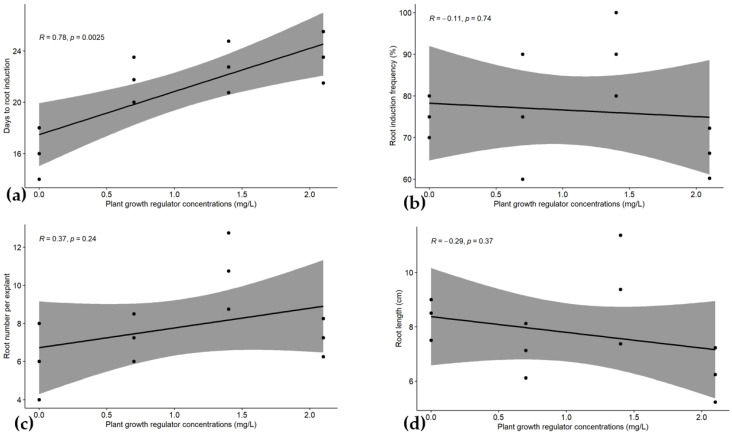
Linear regression curve of plant growth regulator concentrations with days to root formation (**a**), root induction frequency (**b**), root number per explant (**c**), and root length (**d**) in *Musa* spp. R = Pearson correlation (R), calculated using RStudio.

For root induction parameters, a significantly high and positive correlation (r = 0.78) was registered between days to root formation and the IBA concentration at *p* < 0.05 (Figure 13a). However, there was no correlation highlighted for root formation frequency, root length, and root number per plantlet with the PGR concentration at *p* < 0.05 (Figure 13b–d).

## 3. Discussion

In this study, the combination of BAP and IAA or NAA at low concentrations resulted in shoot formation in banana and plantain within a very short period of time. The increase in PGR concentration delayed shoot induction. It has been reported that cytokinin (BAP or BA or Kn) combined with low concentrations of auxins (IAA or NAA) fostered shoot formation in *Solanum tuberosum* and *Althaea officinalis* [23]. However, IBA has less impact on shoot formation, and the downward curve of days to shooting observed would be due to BAP, which reportedly has significant positive effects on banana in vitro shoot induction when applied in high quantities [24]. It was observed that the genome AAA responded faster in shoot formation media compared to AAB and ABB. Other investigations found that the mixed groups (AB, AAB, ABB) of bananas exhibit lower performance compared to the majority of AAA types in in vitro conditions [25].

The shoot induction efficiency and shoot number increased with the increase in BAP concentration when the media contained IAA. Studies have demonstrated that auxins and cytokinins interact in a complex manner to facilitate plant growth and differentiation [26]. It has been reportedly established that exogenous cytokinins, including BAP supplementation under in vitro culture, trigger shoot induction in many cultures at low concentrations, stimulating cell division, bud development, and stem branching [27,28,29,30]. In the same way, Kumar et al. [31] reported that MS medium amended with BAP and IAA was found to be the best combination for shoot induction and elongation for monocots. Other studies highlighted that IAA and BAP, when combined, enhance plant growth, but the mechanism remains unclear [32,33]. The highest shoot formation frequency (100%) and shoot number per explant (7) obtained in this investigation in MS media amended with 3.6 mg.L^−1^ BAP augmented with 0.1 mg.L^−1^ IAA confirms the positive effects of BAP and IAA on shoot induction from regenerated banana and plantain plantlets. These shoot formation data are higher than those recorded in the studies on a micropropagation system for mass clonal production of banana through shoot tip culture with the use of BAP either alone or in combination with NAA [34,35]. This finding suggests that the combination of cytokinin and auxin in shoot formation media is more effective for *Musa* spp. and highlights the importance of optimizing growth media components to enhance shoot formation in banana micropropagation.

In this investigation, the genome AAA exhibited the highest induction frequency, shoot number, and shoot length compared to the other genomes. These results are similar to those of Prabowo et al. [36] and Zhao et al. [37], who found that bananas with the AAA genome had the highest induction rate and shoot multiplication factor among all the tested genomes. Therefore, genome background plays a crucial role in in vitro propagation, as observed in the development of a protocol for propagating *Primula vulgaris*, *Beta vulgaris*, and *Solanum lycopersicum* through shoot regeneration [38,39,40]. It appears that the genotype of a cultivar significantly impacts the shoot proliferation rate, possibly due to cytokinin activity variations in different genotypes, their uptake rates, rates of translocation to meristematic cells, and metabolic processes [41]. However, the statistical analysis of the present study revealed similar shoot formation rates in the genomes AAB and ABB. This result is different from that of Shafira et al. [42], who concluded that the genome ABB adapted faster than AAB on shoot initiation media. The difference with our results could be due to varietal differences.

Root formation was observed in both MS media devoid of PGRs and MS media amended with PGRs. This finding aligns with the study by Ngomuo et al. [43] regarding the effects of auxins on the growth and development of *Musa* spp., in which they noted that in vitro rooting of banana can be effectively achieved by transferring explants to basal media. This output highlights the importance of the growth medium in promoting root development in banana plants, suggesting that the composition of the basal media plays a crucial role in the successful induction of rooting. However, the root induction frequency was lower than that resulting from media containing auxins. Other studies have demonstrated the crucial role played by auxins in enhancing root initiation for plant growth and stability [44]. In addition, the necessity of using auxins for root induction in banana tissue culture is also reported [45]. In media supplemented with PGRs, the two auxin combination types impacted the root formation of the regenerated plantlets differently. Yu et al. [46], in a study on the control of root organogenesis in tissue culture, similarly reported that the auxin behavior on in vitro root induction depends on the type of auxins used. An observation was also made that roots formed in MS media amended with IBA with either NAA or IAA, suggesting that IBA was the key hormone initiating root formation under in vitro conditions. Kollárová’s study [47] on auxins’ impact on *Karwinskia humboldtiana* root cultures revealed that media supplemented with IBA showed optimal adventitious root elongation and lateral root induction. Furthermore, though the number of DRFs increased with increasing IBA concentrations to less than 1.5 mg.L^−1^, the presence of NAA resulted in a greater number of DRFs than IAA. In the same way, the highest root formation frequency and root length were achieved in media amended with IBA combined with IAA. It is well known that IAA, a key substance in the auxin group, significantly influences root formation by stimulating IAA oxidation changes, thus significantly impacting rooting induction formation, especially when associated with IBA [48]. The root initiation frequency (100%) and root number per explant (13 and 8.5) in MS media supplemented with 1.4 mg.L^−1^ IBA combined with 0.5 mg.L^−1^ IAA or NAA, respectively, recorded in this investigation are far higher than those of Arinaitwe et al. [49], Mekonen et al. [50], and Aremu et al. [51]. These optimized results could be explained by the combined effects of IBA and IAA, which appear to promote better root formation in *Musa* spp. under tissue culture conditions. However, the root length observed in this investigation is similar to that of Waman et al. [52].

The three genomes used in this investigation did not show the same dynamic growth in root formation media, highlighting the effects of plant genotypes on in vitro root initiation. The genome AAA performed better and faster than AAB and ABB, producing roots within an average of 8 days in both media, IBA supplemented with IAA or NAA. The influence of genotypes on in vitro rooting for many monocots, including *Saccharum officinarum*, *Manihot esculenta* Crantz, and *Zea mays*, has been reported [53,54]. Furthermore, Yegizbayeva et al.’s study [55] on Persian walnut varieties has revealed that rooting in plants is influenced by genotypes and the culture medium used during propagation. Different genotypes exhibit varying capacities for root development, indicating a genetic basis for rooting success. Additionally, the composition of the culture medium plays a critical role in facilitating or hindering root formation. This interplay between genetic factors and environmental conditions underscores the importance of selecting appropriate genotypes and optimizing culture media to enhance rooting efficiency in plant propagation. Understanding these dynamics can lead to improved practices in horticulture and agriculture. However, the genomes AAB and ABB showed similar rooting potential, with a slight advantage for AAB, confirming the effects of genotypes in tissue culture. In total, the genomes AAA and AAB are identified as the most promising candidates for tissue culture, particularly for genome editing applications, due to their potential for improved and expedited responses. However, other genomes may also be viable options, contingent upon the specific media utilized in the process. This flexibility suggests that while AAA and AAB are preferred, the choice of genome can be adapted based on the experimental conditions and requirements.

The study highlighted a significant positive correlation between the days to shoot formation, shoot induction frequencies, and shoot length with the concentrations of PGRs in banana and plantain tissue culture. This indicates the crucial role that PGRs play in enhancing the growth and development of shoots in these plants. The findings also support the observation that media lacking PGRs resulted in a notably low frequency of shoot formation, underscoring the importance of these regulators in successful tissue culture practices for bananas and plantains [22,56]. The study found no correlation between PGR concentrations and the frequency of root formation, root length, or root number in banana explants. This observation supports previous research indicating that effective in vitro rooting of banana can be achieved by transferring explants to a basal medium, rather than relying on varying PGR concentrations [57,58]. The findings suggest that simpler methods may be sufficient for successful rooting in banana tissue culture.

## 4. Materials and Methods

### 4.1. Plantain and Banana Varieties

Plantain and banana plantlet cultivars were purchased from Alliance of Biodiversity International and CIAT (Europe—Belgium Office) and placed in a CONVIRON growth chamber (Model No. TC80, Serial No. 150423, Winnipeg, MB, Canada) for hardening in the Plant Genomics Laboratory at Bowie State University, Maryland, USA. Plants were transferred to the greenhouse two months later. Four accessions with different genomic backgrounds were used for in vitro regeneration: Gros Michel (AAA genome), Obino l’Ewai and Silk (AAB genome), and Poteau Naine (ABB genome).

### 4.2. Explant Preparation

Healthy, vigorous, and unflowered growing plants of plantain and banana showing no symptoms of infection under greenhouse conditions were collected and used as explants. The in vitro culture was established from the shoots as described by [32] with some modifications. Briefly, the outer layer of leaves and corm tissue was removed from the suckers to obtain an explant of 4–10 cm long. The explants were washed with water for 15 min to remove adherent soils and then subjected to surface sterilization by immersion in 70% ethanol for 12 min and 10% sodium hypochlorite (NaClO) (Fisher Chemical, Pittsburgh, PA, USA) in a closed container for 20 min, respectively. The explants were rinsed 4–5 times with sterilized distilled water. Then, the outer layer of explants was removed. Further, the shoot tip explants were surface sterilized with 5% NaClO containing Tween-20, 2–3 drops for 10 min, then washed 3 times with sterilized distilled water. Another outer layer of the explants was removed carefully. The final size of explants was 1–2 cm, with 2–3 overlapping leaf bases enclosing the auxiliary buds.

### 4.3. Shoot Bud Formation, Multiplication, and Elongation

The 1 cm shoot tip explants were divided into two pieces through the meristem and horizontally cultured under sterile conditions on autoclaved Murashige and Skoog (MS) media [59] containing vitamins (Table A1), 3% (*w*/*v*) sucrose, and 10 mg.L^−1^ ascorbic acid and solidified with 0.44% (*w*/*v*) gelzan (Sigma–Aldrich, St. Louis, MO, USA) for shoot formation. As for the growth hormones, 6-benzyl amino purine (BAP) was supplemented to the medium in varying quantities (1.2, 2.4, 3.6, 4.8, 6.0 mg.L^−1^) in combination with indole butyric acid (IBA, 0.1 mg.L^−1^), indole-3-acetic acid (IAA 0.1, mg.L^−1^), or naphthalene acetic acid (NAA, 0.1 mg.L^−1^). Plant-growth-regulator-free MS medium was used as the negative control. Maintenance of shoot culture was made possible by periodic subculturing on the same medium at a 2-week interval. Cultures were kept at 23 °C ± 2 and 45 µmol·m^−2^s^−1^ light intensity at a 16 h photoperiod, as described by [60]. The same media compositions were considered for shoot multiplication and elongation. At 45-day intervals, the shoot clumps were subcultured on fresh shoot proliferation media. Data recorded included the shoot formation frequency, days to shoot formation, mean shoot length, and mean shoot number per explant.

### 4.4. Root Formation

The shoots emerging from the explants in shoot multiplication media were detached and transferred to root formation media. Rooting of the microshoots was tested using half-strength MS media [1.5% (*w*/*v*) sucrose, 0.22% (*w*/*v*) gelzan] with 10 mg.L^−1^ ascorbic acid, supplemented with IBA (0.7, 1.4, 2.1 mg.L^−1^) combined with either 0.5 mg.L^−1^ IAA or 0.5 mg.L^−1^ NAA. The control was devoid of plant growth regulators (PGRs). The photoperiod, temperature, and light intensity in the growth chamber were similar to those of shoot bud multiplication and elongation conditions. Six weeks following the transfer to rooting, the root formation rate, mean root number, and mean root length per shoot were scored.

### 4.5. Plantlet Acclimatization

Rooted in vitro plantlets were carefully withdrawn from culture bottles and thoroughly cleaned with distilled water to remove any residues of gelzan. Then, they were planted into plastic pots (6″ × 5″) containing Premium Potting Mix (peat moss added to existing soil, USA) and maintained in a controlled environment (CONVIRON) for 45 days. The pots were covered with 8 cm clear plastic bags, and plantlets were watered every three days with a 16 h photoperiod. After one week, the plastic bags were gradually taken off, allowing plantlets to become acclimatized to the natural environment following the previous study by Hassan [61]. The hardened plantlets were then transferred to the greenhouse into a plastic pot (12″ × 10.5″) containing a mixture of Premium Potting Mix and sand (6:1, *v*/*v*). After 2 weeks in the greenhouse, the plant survival rate was assessed.

### 4.6. Experimental Setup and Statistical Analysis

Experiments were designed using a completely randomized design (CRD). Each treatment was tested on four explants and carried out three times. The data analysis was conducted using SAS (version 9.2, SAS Institute, Cary, NC, USA) and R Studio 4.3.2. Analysis of variance (ANOVA) and the calculation of the coefficient of determination (R^2^) were conducted to determine the significance of variation in plantlet shooting and rooting. The means were separated by Fisher’s Least Significant Difference (LSD) at *p* ≤ 0.05 as described by [62].

## 5. Conclusions

The MS medium amended with 3.6 mg.L^−1^ BAP and 0.1 mg.L^−1^ IAA was found to be the most suitable for the in vitro regeneration of *Musa* triploid cultivars, yielding 100% shoot initiation frequency for the three genomes. The genome AAA produced the highest shoot length (10.25 cm) and shoot number per explant (8.8 shoots/explant)) as well as the maximum root induction rates with the media supplemented with IBA and IAA, indicating its potential for tissue culture compared to AAB and ABB. This reproducible in vitro regeneration protocol is important for the large-scale production and conservation of germplasm, ensuring the preservation of the genetic diversity of bananas and plantains. Additionally, it will allow for the initiation of genetic transformation protocols aimed at creating drought-resistant varieties to mitigate water-deficient climate challenges.

## Figures and Tables

**Table 1:** Efficiency of varying phytohormone combinations on shoot formation and proliferation from *Musa* spp. shoot tip explants irrespective of the genomes.

Sr. No	Plant Growth Regulators	Concentration (mg.L^−1^)	Percentage (%) of Shoot Formation ± S.E.	Shoot Number Per Explant ± S.E.
0	MS basal	0	22.50 ± 1.76 j	2.33 ± 0.13 hg
1	BAP + IBA	1.2 + 0.1	31.00 ± 2.87 i	4.33 ± 0.56 de
2		2.4 + 0.1	32 ± 2.58 i	3.25 ± 0.26 efg
3		3.6 + 0.1	37.50 ± 2.31 h	3.67 ± 0.33 ef
4		4.8 + 0.1	50.00 ± 1.76 g	2.67 ± 0.36 fgh
5		6.0 + 0.1	25.00 ± 3.15 j	2.00 ± 0.17 h
6	BAP + IAA	1.2 + 0.1	83.25 ± 2.27 b	5.33 ± 0.06 cd
7		2.4 + 0.1	85.66 ± 2.46 b	5.67 ± 0.21 bc
8		3.6 + 0.1	100.00 ± 00 a	7.00 ± 0.08 a
9		4.8 + 0.1	66.27 ± 2.83 d	6.67 ± 0.22 ab
10		6.0 + 0.1	60.33 ± 4.91 e	3.00 ± 0.00 fgh
11	BAP + NAA	1.2 + 0.1	37.50 ± 2.38 h	2.00 ± 0.80 h
12		2.4 + 0.1	62.75 ± 3.27 de	2.67 ± 0.66 fgh
13		3.6 + 0.1	75.00 ± 2.24 c	4.33 ± 1.04 de
14		4.8 + 0.1	77.66 ± 2.06 c	3.33 ± 0.08 efg
15		6.0 + 0.1	55.33 ± 3.82 f	3.67 ± 0.60 ef
Mean		-	56.36 ± 13.8	3.87 ± 1.57
F Value		-	271.08	16.95
*p*-value		-	<0.05	<0.05
LSD (0.05)		-	4.16	1.10
CV, %		-	4.45	17.06

Values are the mean ± S.E. of 4 replicates per treatment, and all the experiments were repeated thrice. There is no difference between values with the same letter when compared using Fisher’s Least Significant Difference method. LSD = Least Significant Difference. CV = coefficient of variation.

**Table 2:** Effect of *Musa* spp. genomes on axillary shoot production from shoot tip explants in MS media supplemented with 0.1 mg.L^−1^ IAA combined with BAP concentrations.

Sr. No	Plant Growth Regulators	Concentration (mg.L^−1^)	Genome	Percentage (%) of Shoot Formation ± S.E.	Shoot Number Per Explant ± S.E.
0	MS basal	0.00	AAA	30.00 ± 1.57 g	3.00 ± 0.50 g
1			AAB	17.50 ± 5.01 h	2.00 ± 0.40 h
2			ABB	20.00 ± 3.45 h	2.00 ± 0.55 h
3	BAP + IAA	1.2 + 0.1	AAA	94.00 ± 8.12 a	7.00 ± 0.45 bc
4			AAB	80.00 ± 4.17 bc	5.00 ± 0.15 e
5			ABB	75.00 ± 6.51 cd	4.00 ± 0.33 f
6		2.4 + 0.1	AAA	100.00 ± 0.00 a	7.50 ± 0.28 b
7			AAB	80.00 ± 1.75 bc	5.30 ± 0.66 e
8			ABB	75.00 ± 7.25 cd	4.20 ± 0.75 f
9		3.6 + 0.1	AAA	100.00 ± 0.00 a	8.80 ± 0.80 a
10			AAB	100.00 ± 0.00 a	6.60 ± 0.25 cd
11			ABB	100.00 ± 0.00 a	5.60 ± 0.50 e
12		4.8 + 0.1	AAA	83.00 ± 8.61 b	8.50 ± 0.74 a
13			AAB	55.00 ± 5.25 ef	6.30 ± 0.90 d
14			ABB	75.00 ± 4.30 cd	5.20 ± 0.38 e
15		6.0 + 0.1	AAA	70.00 ± 7.08 d	5.00 ± 0.51 e
16			AAB	50.00 ± 10.13 f	3.00 ± 0.35 g
17			ABB	60.00 ± 6.50 e	1.00 ± 0.25 i
Mean		-	-	70.25 ± 8.23	5.00 ± 1.51
F Value		-	-	136.37	104.74
*p*-value		-	-	<0.05	<0.05
LSD (0.05)		-	-	6.57	0.62
CV, %		-	-	5.66	7.58
R^2^		-	-	0.09	0.04

Values are the mean ± S.E. of 4 replicates per treatment, and all the experiments were conducted thrice. No difference between values with the same letter when compared with Fisher’s Least Significant Difference method.

**Table 3:** Effect of *Musa* spp. genomes on axillary shoot production from shoot tip explants in MS media supplemented with 0.1 mg.L^−1^ NAA combined with BAP concentrations.

Sr. No	Plant Growth Regulators	Concentration (mg.L^−1^)	Genome	Percentage (%) of Shoot Formation ± S.E.	Shoot Number Per Explant ± S.E.
0	MS basal	0.00	AAA	30.00 ± 6.00 h	3.00 ± 0.25 e
1			AAB	17.50 ± 1.89 i	2.00 ± 0.13 f
2			ABB	20.00 ± 3.25 i	2.00 ± 0.22 f
3	BAP + NAA	1.2 + 0.1	AAA	45.00 ± 4.11 f	2.00 ± 0.25 f
4			AAB	32.50 ± 2.08 gh	2.00 ± 0.46 f
5			ABB	35.00 ± 3.27 gh	2.00 ± 0.22 f
6		2.4 + 0.1	AAA	72.50 ± 3.86 bc	3.00 ± 0.14 e
7			AAB	55.00 ± 3.45 e	2.00 ± 0.35 f
8			ABB	60.00 ± 3.57 de	3.00 ± 0.42 e
9		3.6 + 0.1	AAA	100.00 ± 0.00 a	5.00 ± 0.41 a
10			AAB	57.50 ± 6.25 e	3.50 ± 0.50 d
11			ABB	67.50 ± 5.16 cd	4.50 ± 0.33 b
12		4.8 + 0.1	AAA	100.00 ± 0.00 a	4.00 ± 0.16 c
13			AAB	60.00 ± 4.61 de	4.00 ± 0.44 c
14			ABB	72.50 ± 5.52 bc	2.00 ± 0.08 f
15		6.0 + 0.1	AAA	80.00 ± 4.63 b	4.00 ± 0.37 c
16			AAB	40.00 ± 5.06 fg	4.00 ± 0.18 c
17			ABB	45.00 ± 3.35 f	3.00 ± 0.28 e
Mean		-	-	55.00 ± 14.42	3.06 ± 0.39
F Value		-	-	58.35	49.25
*p*-value		-	-	<0.05	<0.05
LSD (0.05)		-	-	9.14	0.41
CV, %		-	-	10.04	8.18
R^2^		-	-	0.35	0.36

Values are the mean ± S.E. of 4 replicates per treatment, and all the experiments were conducted thrice. No difference between values with the same letter when compared with Fisher’s Least Significant Difference method.

**Table 4:** Effect of *Musa* spp. genomes on axillary shoot production from shoot tip explants in MS media supplemented with 0.1 mg.L^−1^ IBA combined with BAP concentrations.

Sr. No	Plant Growth Regulators	Concentration (mg.L^−1^)	Genome	Percentage (%) of Shoot Formation ± S.E.	Shoot Number Per Explant ± S.E.
0	MS basal	0.00	AAA	30.00 ± 1.75 f	3.00 ± 0.25 d
1			AAB	17.50 ± 3.88 h	2.00 ± 0.50 e
2			ABB	20.00 ± 2.23 h	2.00 ± 0.25 e
3	BAP + IBA	1.2 + 0.1	AAA	37.50 ± 4.33 c	5.00 ± 0.75 b
4			AAB	25.50 ± 1.74 g	3.00 ± 0.33 d
5			ABB	30.00 ± 5.16 f	5.00 ± 0.66 b
6		2.4 + 0.1	AAA	38.50 ± 6.45 c	5.00 ± 0.21 b
7			AAB	26.00 ± 5.25 g	2.00 ± 0.44 e
8			ABB	31.50 ± 4.26 ef	3.00 ± 0.12 d
9		3.6 + 0.1	AAA	45.00 ± 5.00 b	5.50 ± 0.25 a
10			AAB	33.00 ± 9.16d e	2.25 ± 0.78 e
11			ABB	34.50 ± 2.84 d	3.25 ± 0.55 d
12		4.8 + 0.1	AAA	57.50 ± 1.55 a	4.00 ± 0.37 c
13			AAB	37.50 ± 4.85 c	2.00 ± 0.38 e
14			ABB	55.00 ± 6.12 a	2.00 ± 0.75 e
15		6.0 + 0.1	AAA	33.00 ± 3.93 de	2.00 ± 0.50 e
16			AAB	17.50 ± 4.25 h	2.00 ± 0.29 e
17			ABB	24.50 ± 5.02 g	2.00 ± 0.70 e
Mean		-	-	33.00 ± 7.05	3.06 ± 0.95
F Value		-	-	123.80	78.55
*p*-value		-	-	<0.05	<0.05
LSD (0.05)		-	-	2.89	0.41
CV, %		-	-	5.30	8.18
R^2^		-	-	0.11	0.06

Values are the mean ± S.E. of 4 replicates per treatment, and all the experiments were conducted thrice. No difference between values with the same letter when compared with Fisher’s Least Significant Difference method.

**Table 5:** Effect of varying concentrations of IBA with 0.5 mg.L^−1^ IAA or NAA on *Musa* spp. in vitro rooting from regenerated plantlets irrespective of the genomes.

Sr. No	Plant Growth Regulators	Concentration (mg.L^−1^)	Percentage (%) of Root Induction ± S.E.	Root Length (cm) ± S.E.
0	MS basal	0	75.00 ± 2.00 d	8.50 ± 0.75 b
1	IBA + IAA	0.7 + 0.5	87.50 ± 1.00 b	8.13 ± 0.55 bc
2		1.4 + 0.5	100.00 ± 0.00 a	11.50 ± 0.51 a
3		2.1 + 0.5	65.00 ± 0.20 ef	7.10 ± 0.13 cd
1	IBA + NAA	0.7 + 0.5	62.50 ± 1.66 f	6.13 ± 0.95 de
2		1.4 + 0.5	80.00 ± 1.27 c	7.25 ± 1.67 bcd
3		2.1 + 0.5	67.55 ± 2.53 e	5.38 ± 0.36 e
Mean		-	76.78 ± 13.52	7.71 ± 1.99
F Value		-	106.80	19.82
*p*-value		-	<0.05	<0.05
LSD (0.05)		-	3.97	1.35
CV, %		-	2.95	10.03
R^2^			0.02	0.05

Values are the mean ± S.E. of 4 replicates per treatment, and all the experiments were conducted thrice. No difference between values with the same letter when compared with Fisher’s Least Significant Difference method.

**Table 6:** Effect of *Musa* spp. genomes on in vitro rooting of plantlets in MS media formulation with 0.5 mg.L^−1^ IAA combined with IBA concentrations.

Sr. No	Plant Growth Regulators	Concentration (mg.L^−1^)	Genome	Percentage (%) of Root Formation ± S.E.	Root Length (cm) ± S.E.
0	MS basal	0.00	AAA	80.00 ± 1.57 bcd	10.70 ± 1.70 bc
1			AAB	75.00 ± 5.01 cd	6.60 ± 0.24 gh
2			ABB	70.00 ± 3.45 d	8.20 ± 0.18 ef
3	IBA + IAA	0.7 + 0.5	AAA	90.00 ± 8.12 ab	11.15 ± 1.61 b
4			AAB	87.50 ± 4.17 abc	6.10 ± 0.42 gh
5			ABB	85.00 ± 6.51 bc	7.10 ± 0.32 fg
6		1.4 + 0.5	AAA	100.00 ± 0.00 a	12.90 ± 1.53 a
7			AAB	100.00 ± 0.00 a	9.80 ± 0.35 cd
8			ABB	100.00 ± 0.00 a	11.80 ± 0.47 ab
9		2.1 + 0.1	AAA	100.00 ± 0.00 a	9.30 ± 1.10 de
10			AAB	50.00 ± 2.75 e	5.70 ± 0.36 h
11			ABB	45.00 ± 2.84 e	6.30 ± 0.23 gh
Mean		-	-	81.88 ± 9.33	8.80 ± 2.02
F Value		-	-	17.00	37.53
*p*-value		-	-	<0.05	<0.05
LSD (0.05)		-	-	13.48	1.18
CV, %		-	-	9.77	7.95
R^2^		-	-	0.01	0.05

Values are the mean ± S.E. of 4 replicates per treatment, and all the experiments were conducted thrice. No difference between values with the same letter when compared with Fisher’s Least Significant Difference method.

**Table 7:** Effect of *Musa* spp. genomes on in vitro rooting of plantlets in MS media formulation with 0.5 mg.L^−1^ NAA combined with IBA concentrations.

Sr. No	Plant Growth Regulators	Concentration (mg.L^−1^)	Genome	Percentage (%) of Root Formation ± S.E.	Root Length (cm) ± S.E.
0	MS basal	0.00	AAA	80.00 ± 8.75 ab	10.70 ± 0.55 a
1			AAB	75.00 ± 10.13 abc	6.60 ± 1.35 cde
2			ABB	70.00 ± 9.31 bcde	8.20 ± 1.43 b
3	IBA + NAA	0.7 + 0.5	AAA	75.00 ± 5.16 abc	7.80 ± 0.77 bc
4			AAB	55.00 ± 6.50 f	4.10 ± 0.64 fg
5			ABB	57.50 ± 2.75 ef	6.45 ± 1.18 cde
6		1.4 + 0.5	AAA	87.50 ± 11.02 a	8.25 ± 0.41 b
7			AAB	72.50 ± 8.45 bcd	6.30 ± 1.33 de
8			ABB	80.00 ± 7.26 ab	7.20 ± 0.67 bcd
9		2.1 + 0.1	AAA	77.50 ± 5.67 abc	7.20 ± 1.24 bcd
10			AAB	60.00 ± 3.45 def	3.40 ± 0.51 g
11			ABB	65.00 ± 2.33 cdef	5.50 ± 1.22 ef
Mean		-	-	71.25 ± 7.68	6.81 ± 1.69
F Value		-	-	5.19	15.52
*p*-value		-	-	<0.05	<0.05
LSD (0.05)		-	-	12.86	1.44
CV, %		-	-	10.70	12.55
R^2^		-	-	0.002	0.21

Values are the mean ± S.E. of 4 replicates per treatment, and all the experiments were conducted thrice. No difference between values with the same letter when compared with Fisher’s Least Significant Difference method.

## Data Availability

All data produced and examined throughout this research are included in this article.

## Appendix A

**Table A1:** Murashige & Skoog medium containing vitamins.

Element	Concentration	
	Mg.L^−1^	µM
Microelements		
CoCl_2_.6H_2_O	0.025	0.11
CuSO_4_.5H_2_O	0.025	0.10
FeNaEDTA	36.70	100.00
H_3_BO_3_	6.20	100.27
KI	0.83	5.00
MnSO_4_.H_2_O	16.90	100.00
Na_2_MoO_4_.2H_2_O	0.25	1.03
ZnSO_4_.7H_2_O	8.60	29.91
Macroelements		
CaCl_2_	332.02	2.99
KH_2_PO_4_	170.00	1.25
KNO_3_	1900.00	18.79
MgSO_4_	180.54	1.50
NH_4_NO_3_	1650.00	20.61
Vitamins		
Gly	2.00	26.64
C_6_H_12_O_6_	100.00	554.94
C_6_H_5_NO_2_	0.50	4.06
C_8_H_12_ClNO_3_	0.50	2.43

Source from ref. [63].
